# Influence of Pig Farming on the Human Nasal Microbiota: Key Role of Airborne Microbial Communities

**DOI:** 10.1128/AEM.02470-17

**Published:** 2018-03-01

**Authors:** Julia G. Kraemer, Alban Ramette, Suzanne Aebi, Anne Oppliger, Markus Hilty

**Affiliations:** a Institute for Infectious Diseases, University of Bern, Bern, Switzerland; b Institute for Work and Health, University of Lausanne and University of Geneva, Epalinges, Switzerland; University of Helsinki

**Keywords:** humans, pigs, microbial communities, microbial ecology, occupational health

## Abstract

It has been hypothesized that the environment can influence the composition of the nasal microbiota. However, the direct influence of pig farming on the anterior and posterior nasal microbiota is unknown. Using a cross-sectional design, pig farms (*n* = 28) were visited in 2014 to 2015, and nasal swabs from 43 pig farmers and 56 pigs, as well as 27 air samples taken in the vicinity of the pig enclosures, were collected. As controls, nasal swabs from 17 cow farmers and 26 non-animal-exposed individuals were also included. Analyses of the microbiota were performed based on 16S rRNA amplicon sequencing and the DADA2 pipeline to define sequence variants (SVs). We found that pig farming is strongly associated with specific microbial signatures (including alpha- and beta-diversity), which are reflected in the microbiota of the human nose. Furthermore, the microbial communities were more similar within the same farm compared to between the different farms, indicating a specific microbiota pattern for each pig farm. In total, there were 82 SVs that occurred significantly more abundantly in samples from pig farms than from cow farmers and nonexposed individuals (i.e., the core pig farm microbiota). Of these, nine SVs were significantly associated with the posterior part of the human nose. The results strongly indicate that pig farming is associated with a distinct human nose microbiota. Finally, the community structures derived by the DADA2 pipeline showed an excellent agreement with the outputs of the mothur pipeline which was revealed by procrustes analyses.

**IMPORTANCE** The knowledge about the influence of animal keeping on the human microbiome is important. Previous research has shown that pets significantly affect the microbial communities of humans. However, the effect of animal farming on the human microbiota is less clear, although it is known that the air at farms and, in particular, at pig farms is charged with large amounts of dust, bacteria, and fungi. In this study, we simultaneously investigated the nasal microbiota of pigs, humans, and the environment at pig farms. We reveal an enormous impact of pig farming on the human nasal microbiota which is far more pronounced compared to cow farming. In addition, we analyzed the airborne microbiota and found significant associations suggesting an animal-human transmission of the microbiota within pig farms. We also reveal that microbial patterns are farm specific, suggesting that the environment influences animals and humans in a similar manner.

## INTRODUCTION

The human nares are an important niche for bacterial colonization by both pathogens and commensals, and it is one of the main interfaces between the internal body and the external environment. Pig farmers are exposed to a complex and heterogeneous environment, including large amounts of bacteria on a daily basis (1), and swine represent a potential reservoir for many pathogens that can be transmitted to humans, such as Streptococcus suis and Clostridium difficile (2). Also, there is a growing concern regarding the transmission of antibiotic-resistant bacteria, such as methicillin-resistant Staphylococcus aureus (MRSA) at pig farms and at other livestock-associated areas (3–7). A considerable number of studies have been published showing the transmission of these bacteria from pigs to humans (for reviews, see references 2, 8, 9 and 10). However, previous studies mainly focused on the investigation of only one or two bacterial species and were culture dependent, but the overall impact on the entire human microbiota has never been investigated.

A recent study using culture-independent, next-generation sequencing methods investigated 25 households containing 56 pets and 30 humans and revealed that household membership was strongly associated with microbial communities in both humans and pets (11). In another, longitudinal study, evidence for substantial exchanges among human, home, and pet microbiota were shown as well (12). The authors concluded that such interactions could have considerable human and animal health implications. Some studies have also shown that living or working with animals can protect against asthma and atopic diseases due to exposure to specific animal microorganisms (13, 14). However, despite the relevance, the pattern of the microbiota exchange among animals, humans, and the environment in pig farms has never been investigated. The aims of our study were (i) to describe the influence of pig farming on the human nasal microbiota; (ii) to identify the sequence variants (SVs) predominantly shared between pigs, air from the pig enclosures, and pig farmers; (iii) to identify which of the latter were significantly associated with either the posterior or anterior nasal cavities of pig farmers; and (iv) to compare the findings derived by DADA2 with the outputs of the more traditionally used mothur pipeline.

## RESULTS

### Characterization of sampling population and sequence analysis.

Details of the sampling population can be found in Table 1. In total, 28 pig farms were visited, on which one to three pigs (total *n* = 56), one air sample (total *n* = 27), and one to four pig farmers (total *n* = 43) were sampled (Table 1). As a control, individuals who had contact with cows but no contact with pigs (cow farmers, *n* = 17) and individuals without contact with any type of farm animal (“nonexposed” persons, *n* = 26) working in offices were chosen to assess the effect of pig exposure on human nasal microbiota. All individuals were recruited from the same geographical area and were roughly age matched. After exclusion of 17 samples due to PCR amplification issues, 255 samples with a total of 9,692,391 reads were included in our study. The mean number of reads per sample was 38,009 (± a standard deviation of 19,412), ranging from 2,243 to 120,642 reads. The reads were clustered into a total of 13,585 sequence variants (SVs). The sequencing depth was sufficient, as determined by the low slope of the rarefaction curves (see Fig. S1 in the supplemental material).

**TABLE 1:** Characteristics of the study population

Location	No. of sampled individuals	No. of sampled male individuals	No. of sampled smokers	Mean age of sampled individuals^*a*^ (SD)	No. of sampled pigs	Total no. of pigs on farm	No. of air samples (pig barn)
Pig farm							
1	2	2	0	34 (11)	3	920	1
2	1	1	0	65	2	80	1
3	2	1	0	40 (1)	2	350	1
4	1	1	0	33	2	435	1
5	1	1	0	71	2	240	1
6	1	1	0	45	2	80	1
7	1	1	0	54	2	90	1
8	1	1	0	50	2	120	1
9	1	1	0	48	2	590	1
10	1	1	0	32	2	520	1
11	1	1	0	57	2	90	1
12	2	2	2	32 (5)	2	950	1
13	4	2	1	30 (14)	2	600	1
14	1	1	0	49	2	100	1
15	1	1	0	63	2	520	1
16	1	1	0	42	2	280	1
17	2	1	0	53 (1)	2	290	1
18	1	1	0	62	2	320	1
19	2	1	0	44 (30)	2	250	0
20	1	1	0	53	1	130	1
21	2	2	0	48 (11)	2	750	1
22	2	1	0	53 (4)	2	250	1
23	3	2	2	39 (14)	2	1,950	1
24	2	2	2	58 (4)	2	2,700	1
25	1	1	0	49	2	205	1
26	3	2	0	46 (8)	2	270	1
27	1	1	0	62	2	105	1
28	1	1	0	50	2	350	1
Cow farm							
30	4	4	0	37 (14)			
31	9	8	0	38 (14)			
32	1	1	1	54			
33	2	1	1	60 (0)			
34	1	1	1	50			
Nonexposed							
40–65	26 (one per workplace)	24	9	39 (14)			

a The mean age and standard deviation (SD) are given if more than one individual was sampled.

### Pig farming is associated with increased diversity.

**FIG 1:**
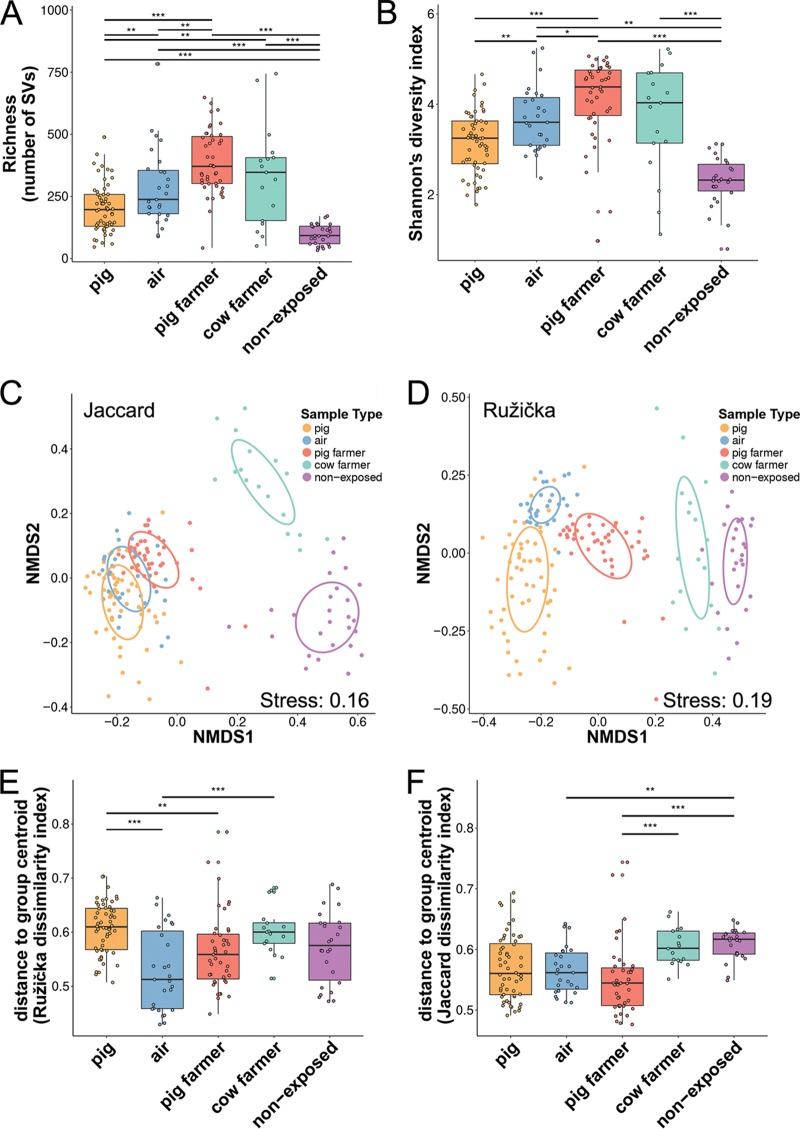
Alpha- and beta-diversity analyses of samples of pigs, air, pig farmers, cow farmers, and non-animal-exposed individuals (“nonexposed”). For the alpha-diversity, the differences in richness (observed SVs) (A) and Shannon diversity indices based on sample types (B) are shown. For the beta-diversity, the unweighted (Jaccard) (C) and weighted (Ružička) (D) distances in the microbiota composition are shown (reduced in a two-dimensional space by NMDS); the 95% confidence ellipse for the group centroid is also shown. (E and F) Beta-dispersion based on Ružička (E) and Jaccard (F) dissimilarity indices in each sample type. The boxplots represent median (midline), interquartile ranges (shaded boxes), and ranges (whiskers). Colors: orange, pig; blue, air; red, pig farmer; green, cow farmer; purple, nonexposed individual. Significant differences within panels A, B, E, and F are indicated by asterisks (*, *P* < 0.05; **, *P* < 0.01; ***, *P* < 0.001).

All 13,585 SVs were grouped into 43 phyla and 310 families and the phyla Actinobacteria, Bacteroidetes, Firmicutes, and Proteobacteria included the majority of all SVs (with an at least 97% mean relative abundance for all sample groups). Pig farmer nasal samples showed the highest Shannon diversity indices (SDIs) and richness, whereas nonexposed samples displayed the lowest SDI and richness values (Fig. 1A and B). To take into account that multiple samples were collected at the same farms, we also performed a linear mixed regression with the location as a random effect to compare the differences between groups. The overall model was significant (analysis of variance; SDI, *P* < 0.001; richness, *P* < 0.001) and showed that the bacterial richness in nasal samples from pig farmers was significantly higher than that of nonexposed individuals (SDI, *P* < 0.001; richness, *P* < 0.001), air samples (SDI, *P* = 0.03; richness, *P* = 0.001), and pig nasal samples (SDI, *P* < 0.001; richness, *P* < 0.001). The alpha-diversity indices in cow farmers were nearly as high as in pig farmers, and the differences were also significant compared to nonexposed samples (SDI, *P* < 0.001; richness, *P* < 0.001) and pig nasal samples (SDI, *P* = 0.003; richness, *P* = 0.002).

### Pig farming influences the microbial community composition.

The ordination method-based nonmetric multidimensional scaling (NMDS) plots with weighted and unweighted input (Fig. 1C and D) showed a distinct clustering of pigs, air, pig farmers, cow farmers, and nonexposed individuals and was confirmed by permutational multivariate analysis of variance (PERMANOVA; unweighted, F-value = 0.15, *P* < 0.001; weighted, F-value = 0.18, *P* < 0.001). Profiles of cow farmers were more similar to nonexposed controls than to pig farmers, indicating a very strong effect of pig farming on the human microbiota. Analysis of similarity (ANOSIM) further confirmed the strong differences between pig farmer and cow farmer/nonexposed samples (see Table S1 in the supplemental material). Interestingly, pig farmers seemed to display a significantly lower beta-diversity dispersion than cow farmers and nonexposed individuals (weighted distances from the centroid; Tukey's honest significant difference [HSD] test; *P* < 0.001; Fig. 1E), indicating that pig farming leads to a more homogeneous microbial community structure. All comparisons of unweighted distances from the centroid were nonsignificant (Tukey's HSD tests; *P* < 0.05; Fig. 1F), suggesting more of an effect of community structure than community composition on variation in beta-diversity across groups of samples.

**FIG 2:**
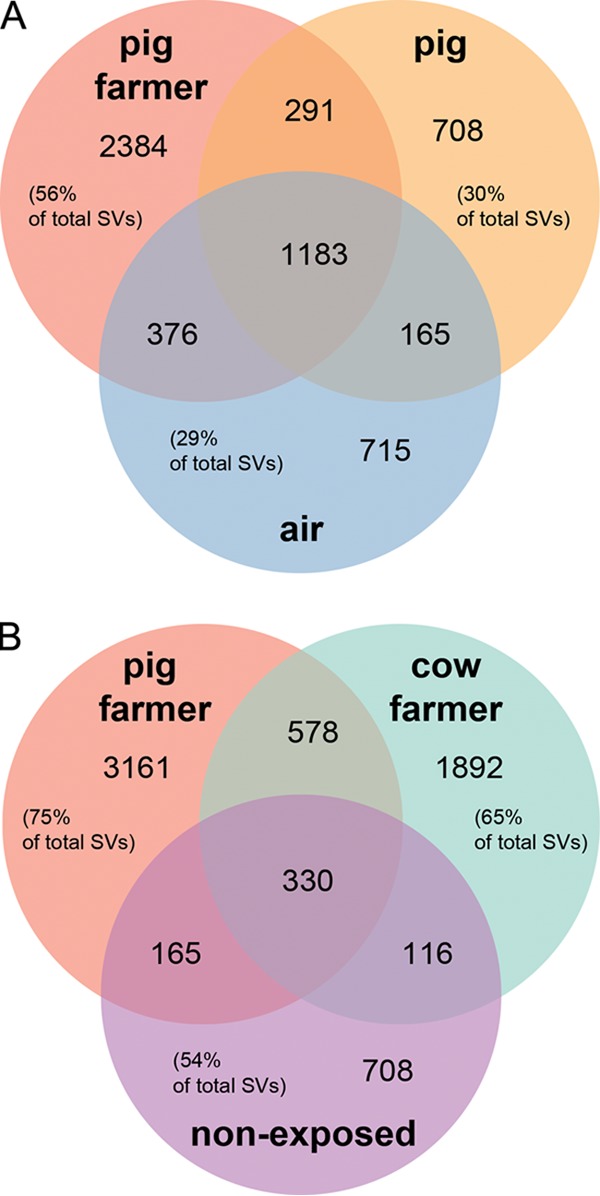
Venn diagram showing unique and shared SVs. (A) Venn diagram showing the numbers of shared SVs between pig farmers, pigs, and air; (B) Venn diagram showing the numbers of shared SVs between pig farmers, cow farmers, and nonexposed individuals. Shared SVs were determined by identifying the total number of shared SVs between pig farmer, pig, and air samples and between pig farmer, cow farmer, and nonexposed samples. Pig farmers share more SVs with pigs and air than with cow farmers and nonexposed individuals.

We next examined how many SVs were shared between sample types; 54% of all SVs occurring in pig farmers also occurred in pigs and/or air, whereas only 25% of the SVs from pig farmers were shared with cow farmers and/or nonexposed individuals (Fig. 2). This illustrates that more SVs are shared within the same environment (pig farms) of the different sample types (pigs, air and pig farmers) than within the same sample type (humans) of the different environments (pig farms, cow farms, and offices).

### The within-farm versus between-farm dissimilarity is reduced.

**FIG 3:**
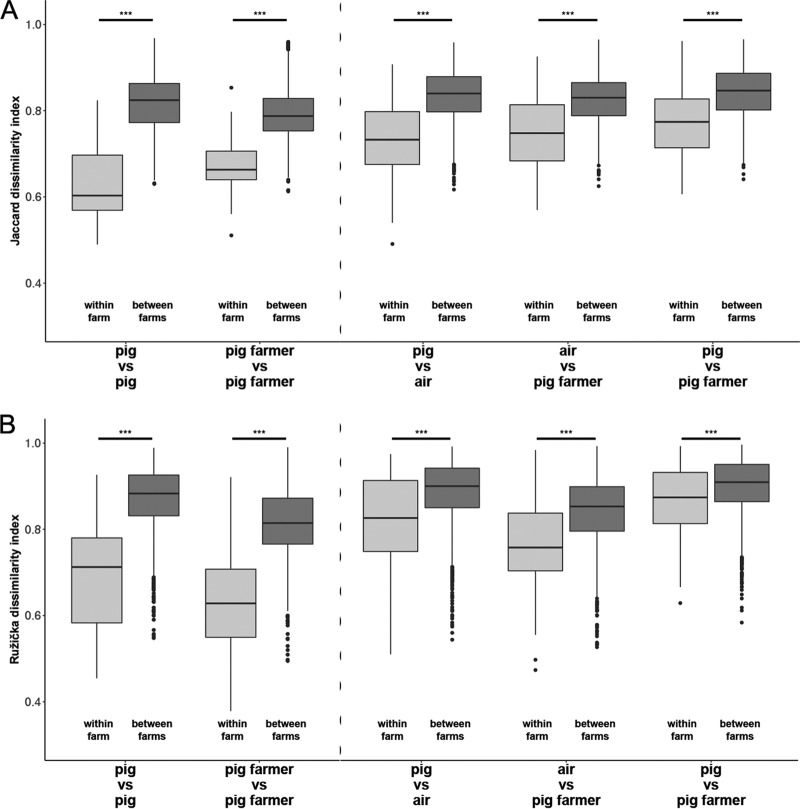
Within- and between-pig-farm dissimilarity measurements. (A and B) Unweighted (Jaccard) (A) and weighted (Ružička) (B) distances in microbiota composition within farms (pairwise distances between sample types originating from the same farm) and in between-farm dissimilarities (pairwise distances between samples originating from different farms). The boxplots show the medians (midline), interquartile ranges (shaded boxes), and ranges (whiskers). Significant differences are indicated by asterisks (*, *P* < 0.05; **, *P* < 0.01; ***, *P* < 0.001). The dotted lines separate comparisons within and between sample types.

In order to investigate whether the microbiota in pig farm samples is influenced by the farm (i.e., the location identification [ID]), we compared pairwise distances between samples originated from the same farm (within farm) and between samples originating from different farms (between farms) (Fig. 3). All “within” distances were significantly lower than the “between” distances (Kruskal-Wallis rank sum tests with the Benjamini-Hochberg [BH] correction [15]; all *P* < 0.001), strongly indicating the existence of an effect of farm characteristics on the microbiota. This was true within (Fig. 3, left side of the dotted line) but also between (Fig. 3, right side of the dotted line) different sample types. However, as expected, the values for the “within” dissimilarities for a given host (Fig. 3, pigs versus pigs and pig farmers versus pig farmers) were generally smaller than the values observed between sample types.

### Identification of SVs significantly associated with pig farming (core pig farm microbiota).

**FIG 4:**
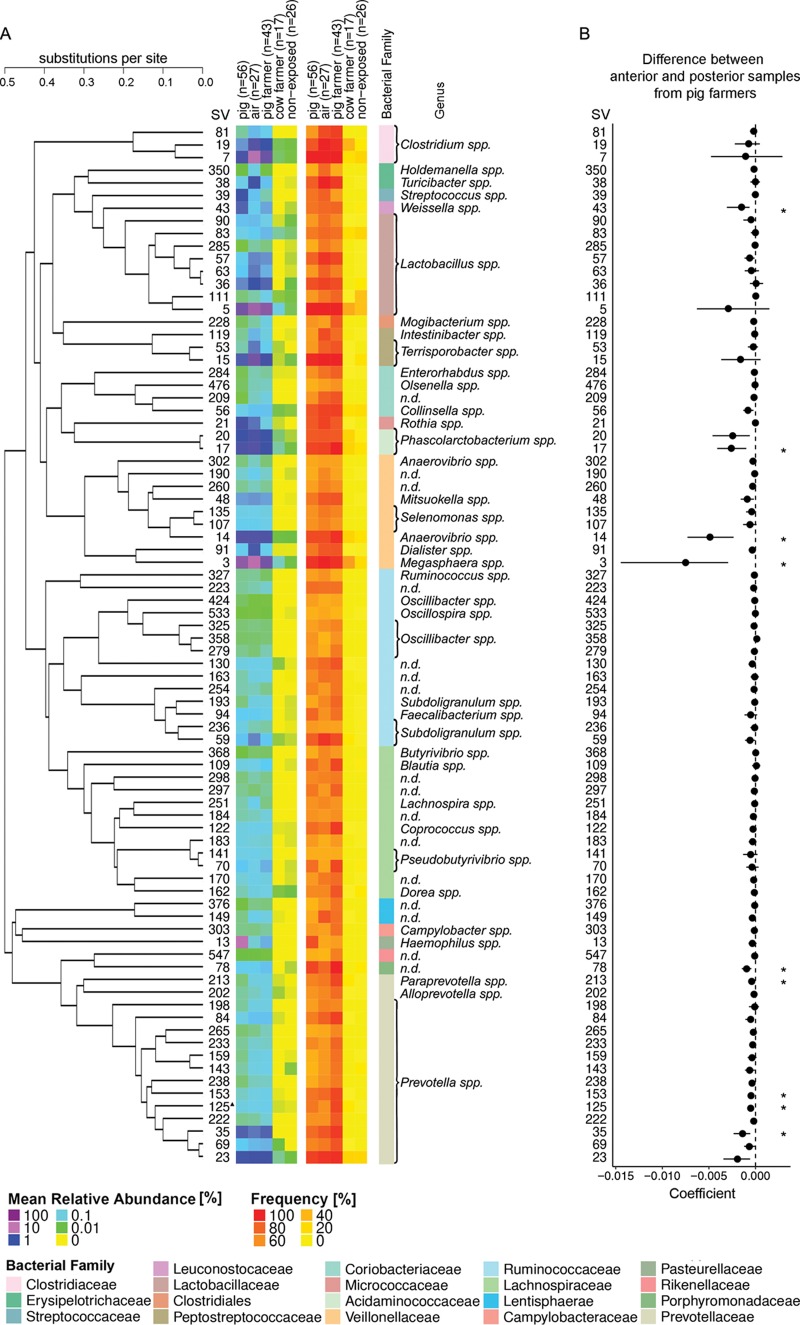
SVs associated with pig farming and differential SVs between anterior and posterior nasal samples. A total of 82 SVs were significantly associated with pig farming (see the text for details). (A) Phylogenetic tree based on differences in SV sequence reads (the distance is displayed as substitutions per site) and heat maps depicting relative abundances and frequencies for pig (*n* = 56), air (*n* = 27), pig farmer (*n* = 56), cow farmer (*n* = 17), and nonexposed individuals (*n* = 26). The assigned taxonomy (bacterial genus, order, or family) for each SV is also indicated. (B) A forest plot displays the coefficients of pairwise differences between anterior and posterior nasal samples from pig farmers derived using Wilcoxon signed-rank tests, followed by the BH correction. Significant differences after multiple testing are indicated by an asterisk (*).

Performing an omnibus test (PERMANOVA) with all factors and all samples (*n* = 255) revealed overall significant factor effects on community variation (*P* = 0.001, with or without stratifying for farm ID). Thus, we next analyzed the SVs that were associated with the changes performing three different analyses. First, SVs that were significantly associated with samples from pig farms were identified by screening all SVs for significantly higher abundance in pigs, air, and pig farmers versus cow farmers, by applying pairwise Mann-Whitney-Wilcoxon tests, followed by the BH correction for multiple testing (15). A total of 82 SVs were identified with significantly higher abundance in samples from pig farms compared to cow farmers, and this low abundance was also present in nonexposed individuals (Fig. 4A). Second, we conducted a similar approach using frequency (presence-absence) data as input, using the Fisher exact test with a BH correction. Eighty-one SVs were identified in both approaches, and one SV (SV125) was identified only by the approach based on relative abundances (see Table S2 in the supplemental material). Finally, we performed an analysis of variance (ANOVA)-like differential expression (ALDEx) analysis for the proportional data (16, 17). Effect size plots showing the “within” and “between” differences for SVs between the respective groups are presented in Fig. S2A to C in the supplemental material. Overall, 41 SVs (50%) were significant for all three analyses, and 9SVs were newly identified using ALDEx (see Fig. S3 and Table S2 in the supplemental material).

### Differences and similarities of the microbiota between anterior and posterior nasal swab samples.

After having identified large microbiota differences in the anterior nasal cavities associated with pig farming, we subsequently analyzed whether there were also associations with the posterior part of the nose. For this, we again first performed an omnibus test (PERMANOVA, nested per individual) with all SVs from pig farmer samples (*n* = 86) that showed an overall significance (*P* = 0.001) between anterior and posterior in each individual. We next analyzed all the 82 SVs that were identified as being specific for pig farming. In total, 9 of 82 SVs were significantly more abundant in the posterior than in the anterior part of the nose, and these included SVs from the bacterial families of Prevotellaceae and Veillonellaceae (Wilcoxon signed-rank tests with the BH correction [15]) (Fig. 4B; *P* < 0.05). We then analyzed the ten most abundant SVs (see Fig. S4 in the supplemental material), and predominantly SVs from Corynebacteriaceae and Staphylococcaceae were more frequently found in the anterior than in the posterior part of the nose (see Fig. S4 in the supplemental material).

### Analysis of sequencing data using the mothur pipeline.

**FIG 5:**
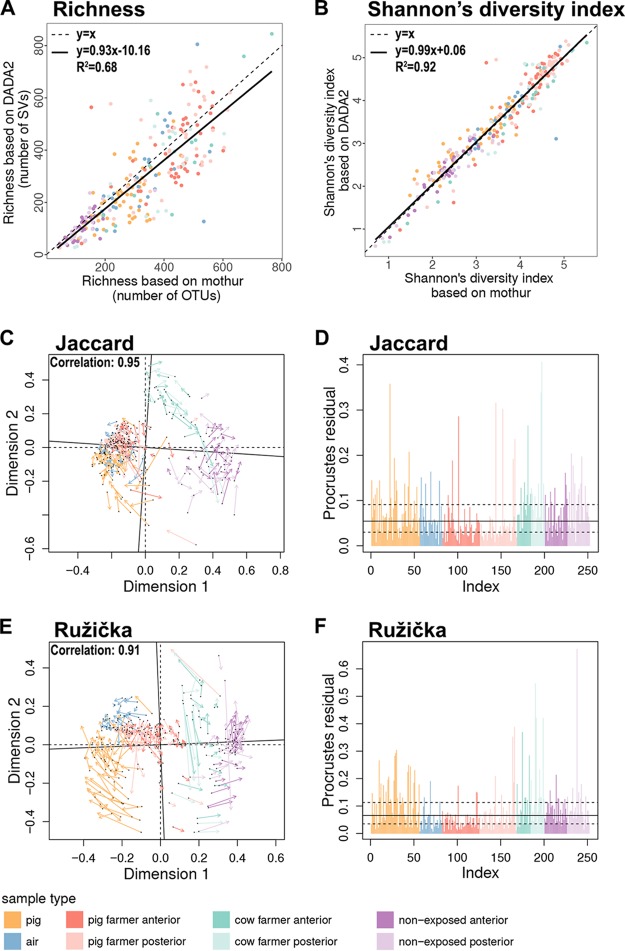
Alpha- and beta-diversity comparisons calculated using DADA2 and mothur. (A) Correlation analysis of richness values; (B) correlation analysis of SDIs; (C) procrustes analysis of Jaccard dissimilarity values (the significant [*P* = 0.001] correlation value is indicated in the figure); (D) bar chart of procrustes residuals based on the Jaccard dissimilarity; (E) procrustes analysis of Ružička dissimilarity (the significant [*P* = 0.001] correlation value is indicated in the figure); (F) bar chart of procrustes residuals based on Ružička dissimilarity.

Finally, we compared our findings from the DADA2 with the mothur pipeline. As for mothur, the final mean number of reads per sample was 34,232 (95% confidence interval = ±2,117) ranging from 3,340 to 109,182 reads, and the sequences were clustered into a total of 31,951 operational taxonomic units (OTUs). After rarefying, 10,553 OTUs were left with 3,340 reads per sample. These OTUs clustered into 41 phyla and 310 families, respectively. The taxonomic profiles were very similar to the profiles obtained with DADA2 (see Fig. S5A to D in the supplemental material), except for a slightly higher abundance of “others” for the samples analyzed using mothur. We also noted a very high correlation between DADA2 and mothur in the case of alpha- and beta-diversity. Richness (*R*^2^ = 0.68) and SDI (*R*^2^ = 0.92) showed strong positive linear relationships between values based on DADA2 and mothur (Fig. 5A and B). The Procrustes analysis comparing beta-diversity values from these two pipelines (Fig. 5C to F) also showed a strong correspondence between these two data sets for both Jaccard and Ružička dissimilarity (procrustes symmetric correlation: Jaccard, 0.95, *P* = 0.001; Ružička, 0.91, *P* = 0.001). The number of procrustes residuals were evenly distributed between the investigated sample types (Fig. 5D and F).

## DISCUSSION

This cross-sectional study investigated the relationships between pig farming and the composition of the nasal microbiota of farmers. We revealed an increased bacterial richness and diversity in the anterior nose of pig farmers versus cow farmers and a nonexposed control group. In addition, beta-diversity analyses revealed significant differences in the composition of the nasal microbiota of these human groups. Samples from within the pig farms shared more of their microbiota compared to the samples from between farms. We were also able to identify the SVs that were significantly associated with pig farming and the SVs that were predominantly more abundant in the posterior than in the anterior nasal cavities of the pig farmers.

These differences in alpha-diversity suggest that farmers raising pigs have an increased bacterial diversity in their noses compared to nonexposed individuals and farmers working on a cow farm. A possible explanation for this is that the high concentration of diverse aerosolized bacteria present in pig barns leads to a modification and an enrichment of the “natural” farmer's nasal microbiota. Therefore, it appears that the establishment of this modified microbial community could be a “fingerprint” of the nasal microbiota of pig farmers. As for changes in community structure (beta-diversity), we revealed that samples from pigs, air, and pig farmers form distinct, yet related clusters, which are all clearly separated from samples from cow farmers and nonexposed office workers. It becomes obvious from our data that pig farming is associated with stronger divergence of the human nasal microbiota compared to cow farming. These findings could be explained by the fact that pig farmers spend more time in a confined environment with the animals than cow farmers and by the fact that airborne dust concentration are higher in pig than in cow farms (18). It has been shown that pets can share a small part of their microbiota with their owners by hypothesized, frequent direct contacts (11, 19). However, our study data strikingly points out that airborne microbiota may indeed play an important role in this microbial transfer. Moreover, we show that the extent of microbiota sharing between pigs and farmers is remarkable.

We also found that samples from farmers working on the same farm shared more of their microbiota than they do with individuals from different farms. This was true not only for pig farmers but also when comparing air samples and pigs from the same farm, hinting at the existence of an even more pronounced farm-specific microbiota. Similarly, it has been shown that household members shared more of their microbiota than they do with individuals from different households (12, 19). In our study, the degree of shared microbiota was large and the type of farm management practices could be influential. Indeed, it has been already shown that farm management (diet and antimicrobial use) influences the nasal microbiota of pigs (20). Therefore, we can hypothesize that the management and the farm characteristics can also have an influence on the air quality of the barn. Humans inhale 10,000 liters of air per day, and airborne bacteria may have a direct effect on the nasal bacterial communities of humans (21). Furthermore, it is known that the air on farms, and in particular on pig farms, is charged with large amounts of dust, bacteria, and fungi (and in other studies [22, 23]) and that the concentration of airborne bacteria can be 2 × 10^7^ times higher than the level usually measured in indoor air (24).

Our results strikingly revealed a very high number of SVs shared between the pigs, air, and the pig farmers, indicating a frequent exchange of members of the microbiota and suggesting that air could play an important role in the transmission of animal-associated bacteria to the farmers, too. Among these SVs, Veillonellaceae and Lactobacillaceae were the most abundant groups in pigs, air, and pig farmers. Lactobacillaceae and Veillonellaceae have been found in the nares of both healthy pigs and humans (20, 25–27). We also simultaneously sampled the posterior and anterior nasal cavities of the human participants. This is important since spatial variation in nasal microbial communities has been highlighted (28), although, in another study, the bacterial composition did not significantly change along the nasal passage (29). In addition, the microbiota of the posterior cavity should reflect a more persistent (versus transient) colonization than that of the anterior cavity. Our data show that the microbiota differ between anterior and posterior nasal cavities and that there are some SVs which are associated with either of the two sites.

The farmer's respiratory tract also receives a lot of attention due to the hygiene hypothesis demonstrating that growing up on a farm may be protective against allergies and asthma (13). This has been shown to be very significant in the case of pig farming (30). Therefore, SVs associated with pig farming identified in this study that were found in higher abundance in the posterior region of the nose could hypothetically be protective against asthma development. Indeed, many SVs found in our study have been associated with respiratory health rather than disease, such as asthma (31–34). Therefore, these SVs could have potential protective implication for allergic and atopic diseases. However, since we only included healthy adult subjects, differences in the nasal microbiota that were associated with certain occupational health problems and/or health benefits (e.g., atopic and allergic diseases) were not investigated.

Within this study, we decided to use the DADA2 algorithm rather than the better known 97% OTU approach. The DADA2 algorithm has been shown to produce a higher resolution of microbial populations when applied to 16S rRNA gene sequences than the popular clustering into OTUs implemented in the mothur or QIIME pipelines (35). The resulting SVs only contain one read per SV, making additional analysis steps, such as oligotyping, unnecessary (36, 43). Even though DADA2 leads to a decrease in alpha-diversity, it does not lead to changes in the community structure, which makes the approach comparable to results produced by other clustering algorithms (35, 37). By comparing DADA2 to mothur in our study, we can clearly confirm the later statement, as shown in our procrustes analyses.

This study has some major strengths. Taking into account all potential confounding factors (season, age, and geographical region), we reliably demonstrated that pig farming has an extensive effect on the human nasal microbiota, and we were able to show the specific SVs that were associated with these changes. Moreover, recruiting cow farmers as a control group allowed us to ascertain that the observed differences are linked to close contact to pigs and not simply to the lifestyle associated with living on a farm. By including multiple samples from identical farms, we were also able to demonstrate the existence of a pronounced farm-specific microbiota by observing more similarity between the microbiota within the same farm than between different farms. Finally, we also included microbiota analysis of posterior nasal samples, and bacteria from this region of the nose are more likely to be relevant for the respiratory tract microbiota and community disturbance that could lead to potential dysbiosis (34, 38).

There are limitations to this study, too. We only included healthy adult subjects. We were thus not able to investigate differences in the nasal microbiota that were associated with certain occupational health problems (e.g., atopic and allergic diseases). Therefore, the relevance of the distinct microbiota needs to be studied in the future with different experimental designs. In addition, we did not perform longitudinal sampling, and therefore we were not able to investigate the temporal stability of the different microbiota. Finally, since this was a “field study,” we did not perform some additional upper or even lower respiratory tract sampling. This would more clearly have shown the composition of the respiratory tract microbiota, as demonstrated previously (32, 33).

In conclusion, we show here that pig farming has an extensive effect on the human nasal microbiota, and we were able to reveal the specific SVs associated with these changes. The relevance and stability of these changes need to be investigated in future studies.

## MATERIALS AND METHODS

### Study design and sampling.

Ethical clearance for this study was obtained from the Human Research Ethics Committee of the Canton Vaud (243/14 and P_2017-00265) and the Veterinary Ethics Committee of the Canton Vaud (VD2903). Sample collection was conducted between October 2014 and March 2015 in the western part of Switzerland. We focused on the winter season, since we hypothesized that doors, etc., may be more likely to be closed and thus the pig farmers are more exposed to indoor bacterial communities. Related to this, it has been described that there is a decrease in some of the air contaminants during summer of swine confinement buildings. In total, 28 pig farms were visited, and nasal swabs from suckling or weaning pigs were obtained by swabbing their noses using sterile cotton swabs. Piglets rather than pigs were chosen for ease of handling and sampling. The pig farmers collected two swabs, outside the pig barn, from their left nares (anterior and posterior), themselves under supervision of the study personnel. In addition, personal information was collected in a questionnaire. Airborne bacteria were sampled with a Coriolis μ air sampler (Bertin Technologies, Montigny-le-Bretonneux, France), positioned approximately 1 m above ground in the middle of the pig house, and airborne particles sampled from 3 m^3^ of air (0.3 m^3^/min for 10 min) were collected into a sterile cone containing 15 ml of 0.005% Triton X-100 solution. As controls, 17 cow farmers and 26 nonfarming individuals, having no contact with any type of farm animal, were included. All samples were immediately transported to the laboratory in a cold box (4°C) and stored at −20°C until further analysis. DNA extraction, amplification, and sequencing were done as outlined in the supplemental material. In brief, the V4 region of the 16S rRNA gene was amplified using forward (5′-GTGCCAGCMGCCGCGGTAA-3′) and reverse (5′-GGACTACHVGGGTWTCTAAT-3′) primers previously described (39) and modified with an Illumina adaptor sequence at the 5′ end. Samples were submitted to a next-generation sequencing platform for indexing and pair-end sequencing (2 × 250 bp; reagent kit, v2) on an MiSeq platform (Illumina, San Diego, CA). Reads were analyzed using the DADA2 package version 1.5.0 and workflow (35) in R version 3.1.2 (http://www.R-project.org) as illustrated in the supplemental material. The output of DADA2 consist of exact SVs that replace the traditional OTUs received by more “traditional” pipelines such as mothur. Using DADA2, no rarefying of sequence reads was necessary.

### Alpha- and beta-diversity analyses and identification of SVs associated with pig farming.

Unless stated otherwise, all calculations were performed in R utilizing functions from R base or the “vegan” package. We did not rarefy our sequences for downstream analyses since the DADA2 algorithm drastically reduces the issues of having different sequencing depths for the samples being compared, which is the main reason for rarefying. Alpha-diversity (the within-sample diversity) was assessed by calculating richness and SDIs, using the functions estimate and diversity. Linear regression models with a random effect to correct for clustering on the location level was used to test for statistical significances between sample types (the lmer function from the lmeTest package), and the overall significance of these models was confirmed by ANOVA (the anova function).

Beta-diversity (the between-sample diversity) was measured by the weighted Ružička index (abundance based) and the unweighted Jaccard index (presence/absence based) of dissimilarity. Ružička is also called the quantitative version of Jaccard and, unlike Bray-Curtis, which is semimetric, is metric and probably should be preferred (http://cc.oulu.fi/~jarioksa/softhelp/vegan/html/vegdist.html). Pairwise distances between samples were calculated using the vegdist function, and the resulting matrices were used to generate NMDS plots (metaMDS function) and dissimilarity boxplots. Significant groupings between samples were assessed by a permutational multivariate analysis of variance using 1,000 Monte Carlo permutation tests (PERMANOVA; adonis function). Analyses of similarities were performed to test for significant differences between groups of samples using 1,000 Monte Carlo permutation tests (ANOSIM; anosim function), followed by the Bonferroni correction for multiple testing. Both PERMANOVA and ANOSIM were performed as hierarchical models with nesting at the farm level to address the fact that several samples originated from the same farm. The extent of beta-diversity dispersion for each sample group was calculated as the average distance (based on the Jaccard and Ružička index) to the sample type's centroid using the betadisper function (40), and significant differences were assessed with the Tukey's HSD test (TukeyHSD function). Significant differences between the groups in the dissimilarity boxplots were assessed by Kruskal-Wallis rank sum tests with the BH correction for multiple testing (15). Boxplots and NMDS plots were generated in R utilizing the ggplot2 package, and Venn diagrams were created with help of the VennDiagram package.

The identification of SVs associated with pig farming and of SVs associated with either anterior or posterior nasal cavities is described in the supplemental material. This includes the ALDEx analysis in R to analyses proportional data using the aldex2 package, as described previously (17).

### Comparison of the pipelines DADA2 and mothur.

We also compared the findings from the DADA2 with the mothur pipeline as illustrated in the supplemental material. In brief, reads of all samples were additionally analyzed using mothur software (v1.36.1) (41) as indicated in the MiSeq standard operating procedure (42). Unlike with DADA2, the data were normalized by random subsampling of sequences resulting in 3,340 reads per sample. Beta-diversity comparison was accomplished by using procrustes transformations with NMDS ordinations (based on Jaccard and Ružička indices of dissimilarity) as the input. The plots were obtained by using the procrustes function and the significance between the two configurations was confirmed with the protest function.

### Accession number(s).

The sequencing reads for this study were deposited at the NCBI Sequence Read Archive under accession no. PRJEB21578.

## Supplementary Material

Supplemental material
