# Supplementary material for: Influence of Pig Farming on the Human Nasal Microbiota: Key Role of Airborne Microbial Communities
Source: Appl Environ Microbiol. 2018 Mar 1;84(6):e02470-17. doi: 10.1128/AEM.02470-17 (PMC5835734; doi:10.1128/AEM.02470-17)
Supplement: Supplemental material [file supp_84_6_e02470-17__index.html]

Supplemental material 

# Influence of Pig Farming on the Human Nasal Microbiota: Key Role of Airborne Microbial Communities

## Supplemental material

**Files in this Data Supplement:**

- Supplemental file 1 -

  Supplemental methods; results of ANOSIM based on Jaccard and Ružička dissimilarity indices (Table S1); significant SVs according to the "abundance-based" approach, presence/absence analysis, and ALDEx analysis (Table S2); rarefaction curves of all the samples included in this study (Fig. S1); effect plots summarizing the ALDEx2 output (Fig. S2); Venn diagram of the three different analyses (Fig. S3); sequence variants (SVs) associated with pig farming and differential SVs between anterior and posterior nasal samples (Fig. S4); taxonomic profile comparison with taxa assignment based on DADA2 and mothur pipelines for all sample types (Fig. S5).

  PDF, 1.3M
