# Supplementary material for: Influence of Pig Farming on the Human Nasal Microbiota: Key Role of Airborne Microbial Communities
Source: Appl Environ Microbiol. 2018 Mar 1;84(6):e02470-17. doi: 10.1128/AEM.02470-17 (PMC5835734; doi:10.1128/AEM.02470-17)
Supplement: Supplemental material [file AEM.02470-17_zam006188389s1.pdf]

Supplementary material (Online Repository)

Supplementary methods: DNA extraction, amplification and sequencing

The nasal swabs were inoculated with 500 µl phosphate-buffered saline (PBS) and vortexed for 15 seconds to transfer the DNA into solution. DNA from air and nasal swabs was extracted using the Qiagen DNA Minikit (Qiagen, Hilden, Germany), following the Spin Protocol for DNA Purification from Body Fluids. From these DNA extracts, the V4 region of the 16S rRNA gene was amplified using forward (5'-GTGCCAGCMGCCGCGGTAA-3') and reverse (5'-GGACTACHVGGGTWTCTAAT-3') primers previously described (1) and modified with an Illumina adaptor sequence at the 5' end. The PCR mix consisted of 21.6 µl molecular grade water, 1x Fast Start Taq reaction buffer, 2 mM magnesium chloride, 0.2 mM deoxyribonucleotide triphosphate, 1 µM of forward and reverse primers, one unit of Fast Start Taq Polymerase (Roche Molecular Biochemicals, Rotkreuz, Switzerland) and 10 µl of extracted DNA, totaling up to a volume of 50 µl. PCR cycling conditions comprised of an initial denaturation at 95 °C for 6 minutes and 35 cycles of denaturation at 95 °C for 30 seconds, annealing at 59 °C for 30 seconds and elongation at 72 °C for 1.5 minutes. This was followed by a final elongation step at 72 °C for 5 minutes. PCR products were purified by QIAquick PCR Purification Kit (Qiagen, Hilden, Germany) and the purified DNA was eluted in 30 µl molecular grade water. The samples were quantified via gel electrophoresis and samples with low DNA concentration were additionally quantified using the DNA 7500 kit with an Agilent 2100 Bioanalyzer (Agilent Technologies, Palo Alto, CA). Samples, taken from individuals with antibiotics intake during the last six months or pig farmers working with pigs for less than six months (two farms), were excluded from this study. As recommended by a previous study, samples below 1 ng/µl after PCR and Purification were excluded from further analyses as well (2). As part of our quality control, a clean cotton swab tip was exposed for several seconds during the sampling procedure and processed together with the samples from this study. Additionally, an extraction control (200 µl PBS) was included for every batch of 60 samples and a PCR control (10 µl sterile water) was included for each amplification

batch to ensure that the used reagents were not resulting in a contamination. However, none of the ‘negative’ control samples were above 1 ng/µl after PCR and Purification and were, therefore, not sent for sequencing. Samples were submitted to the Next Generation Sequencing Platform at the University of Bern for indexing and pair-end 2x250 bp sequencing (Reagent Kit v2) on the Illumina MiSeq platform (San Diego, USA).

Supplementary methods: Analysis of sequencing data using the DADA2 pipeline

Reads were analysed using the dada2 package version 1.5.0 and workflow (3) in R version 3.1.2 (http://www.R-project.org). Forward reads were trimmed at 200 bp and reverse reads were trimmed at 150 bp to remove low quality regions. The 20 first base pairs and instances of a quality score less than or equal to two were truncated from all reads. Reads (and their respective forward or reverse read) containing ambiguous bases and more than two expected errors were filtered out. Then, all reads with identical sequences were collapsed to reduce computational time. The amplicon errors were modeled and corrected using the DADA2 algorithm with default parameters. The denoised output reads were merged and all reads with any mismatches were removed. SVs shorter than 245 or longer than 257 base pairs where removed and chimeras were identified using the *removeBimeraDenovo* function using the pooled method (56.4% of SVs and 8.7% of reads removed). Taxonomy was assigned using the *assignTaxonomy* function, which implements the RDP classifier method (4). A DADA2-formatted training set was used to assign the taxonomy and was derived from Silva version 123 (5). Sequences aligning to chloroplasts, mitochondria, Archaea and Eukaryotes were removed (4.8% of SVs and 4.3% of reads removed).

Supplementary methods: Identification of SVs associated with pig farming

Before investigating the associations of specific SVs, we performed and overall omnibus test (PERMANOVA) with all the factors and all the samples (n=255) with and without stratifying for farm ID to reveal the overall significance. Next, SVs associated with samples from pig farms were obtained by comparing the relative abundance of occurring SVs between the sample group cow farmer and the three sample groups originating from pig farms (pig, air

and pig farmer) with independent Mann-Whitney-Wilcoxon Tests and followed by BH correction (6). Mann-Whitney-Wilcoxon Tests were conducted to compare the relative abundance of each SV between cow farmers and pig farmers followed by a BH correction for multiple testing. This procedure was repeated for the comparison cow farmer - pigs and cow farmer - air. An SV was only chosen to be associated with pig farming if the SV showed a significantly higher abundance in the sample group from pig farms in all the tested comparisons (pig - cow farmer, air - cow farmer and pig farmer- cow farmer). In addition to the Mann-Whitney-Wilcoxon-Test, Fisher's exact tests with an unweighted (presence-absence) input were performed in the same manner to evaluate the differences in occurrence of SVs in pig farming. These two approaches were verified with an ANOVA-Like Differential Expression (ALDEx) Analysis in R using the *aldex2* package. For this, instances of the centered log-ratio transformation values were generated (*aldex.clr* function) and significant differences were assessed. Overall significant differences were investigated via an omnibus test (generalized linear model and Kruskal Wallace tests for one-way ANOVA with BH correction (6); *aldex.glm* function) and significant differences between cow farmers and samples from pig farms (pigs, air and pig farmers) were assessed using Wilcoxon rank tests with BH correction (6)(*aldex.ttest*). The heatmap, displaying the relative abundance and the frequency of the pig farm-associated SVs, was created using the *ComplexHeatmap* and *circlize* packages in R and the phylogenetic tree was calculated using *webPRANK* (7). The effect plots were generated using the *aldex2* package in R (functions *aldex.effect* and *aldex.plot*).

Supplementary methods: Identification of SVs associated with either the anterior or posterior nasal cavities

Paired differences between anterior and posterior nasal samples obtained from pig farmers were investigated for the above mentioned 82 SVs associated with pig farming by calculating Wilcoxon singed rank tests followed by BH correction (6). In addition, we investigated the anterior-posterior nasal cavity differences in pig farmers for the ten most abundant SVs in the

same manner. The graphical visualization of these comparisons was accomplished by using the package *forestplot* in R.

Supplementary methods: Analysis of sequencing data using the mothur pipeline

We also compared the findings from the DADA2 with the Mothur pipeline. For this, reads were additionally analyzed using the mothur software (version 1.36.1) (8) as indicated in the MiSeq standard operating procedure (9). Paired-end reads were aligned and all reads were removed that contained ambiguous bases, stretches of homopolymers longer than eight nucleotides, sequences longer than 254 or shorter than 252 base pairs and sequences that did not align to the target region. Chimeras were identified and removed using UCHIME software (10) and sequences aligning to chloroplasts, mitochondria, Archaea and Eukaryotes were detected and removed as well. Operational taxonomic units (OTUs) were determined with average neighbor algorithm, using a 3% dissimilarity threshold and the taxonomy was assigned using SILVA alignment as a template (5). The data was normalized by random subsampling of sequences resulting in 3340 reads per sample. Subsequently, alpha- and beta-diversity was determined in the same manner as the data obtained with the DADA2 pipeline (see Materials and Methods).

Supplementary methods: Comparison of the pipelines DADA2 and mothur

OTUs and SVs were clustered on family and phylum levels respectively and the taxonomic profiles are shown as mean relative abundance per sample type. The alpha diversity relationship between mothur and DADA2 was evaluated via linear regression (*lm* function). Both stacked bar graphs and scatterplots were produced in R using the *ggplot2* package. Beta-diversity comparison was accomplished by using Procrustes transformations with non-metric multidimensional scaling (NMDS) ordinations (based on Jaccard and Ružička indeces of dissimilarity) as input. The plots were obtained by using the *procrustes* function and the significance between the two configurations was confirmed with the *protest* function.

References

1. Caporaso JG, Lauber CL, Walters WA, Berg-Lyons D, Lozupone CA, Turnbaugh PJ, Fierer N, Knight R. 2011. Global patterns of 16S rRNA diversity at a depth of millions of sequences per sample. Proc Natl Acad Sci U S A 108 Suppl 1:4516-22.
2. Biesbroek G, Sanders EA, Roeselers G, Wang X, Caspers MP, Trzcinski K, Bogaert D, Keijser BJ. 2012. Deep sequencing analyses of low density microbial communities: working at the boundary of accurate microbiota detection. PLoS One 7:e32942.
3. Callahan BJ, McMurdie PJ, Rosen MJ, Han AW, Johnson AJA, Holmes SP. 2016. DADA2: High-resolution sample inference from Illumina amplicon data. Nat Meth 13:581-3.
4. Wang Q, Garrity GM, Tiedje JM, Cole JR. 2007. Naïve Bayesian Classifier for Rapid Assignment of rRNA Sequences into the New Bacterial Taxonomy. Applied and Environmental Microbiology 73:5261-7.
5. Quast C, Pruesse E, Yilmaz P, Gerken J, Schweer T, Yarza P, Peplies J, Glockner FO. 2013. The SILVA ribosomal RNA gene database project: improved data processing and web-based tools. Nucleic Acids Res 41:D590-6.
6. Benjamini Y, Hochberg Y. 1995. Controlling the False Discovery Rate: A Practical and Powerful Approach to Multiple Testing. Journal of the Royal Statistical Society Series B (Methodological) 57:289-300.
7. Loytynoja A, Goldman N. 2010. webPRANK: a phylogeny-aware multiple sequence aligner with interactive alignment browser. Bmc Bioinformatics 11:6.
8. Schloss PD, Westcott SL, Ryabin T, Hall JR, Hartmann M, Hollister EB, Lesniewski RA, Oakley BB, Parks DH, Robinson CJ, Sahl JW, Stres B, Thallinger GG, Van Horn DJ, Weber CF. 2009. Introducing mothur: open-source, platform-independent, community-supported software for describing and comparing microbial communities. Appl Environ Microbiol 75:7537-41.
9. Kozich JJ, Westcott SL, Baxter NT, Highlander SK, Schloss PD. 2013. Development of a dual-index sequencing strategy and curation pipeline for analyzing amplicon sequence data on the MiSeq Illumina sequencing platform. Appl Environ Microbiol 79:5112-20.

10. Edgar RC, Haas BJ, Clemente JC, Quince C, Knight R. 2011. UCHIME improves sensitivity and speed of chimera detection. Bioinformatics 27:2194-200.

Supplementary table S1: Results of ANOSIM based on Jaccard and Ružička dissimilarity indices

compared sample types	R based on Ružička dissimilarity index[a]	p-value	R based on Jaccard dissimilarity index[a]	p-value
Overall	0.58	<0.001	0.577	<0.001
pig - air	0.24	0.003	0.149	<0.001
pig - pig farmer	0.363	<0.001	0.284	<0.001
pig - cow farmer	**0.975**	<0.001	**0.98**	<0.001
pig - non-exposed	**1**	<0.001	**0.989**	<0.001
air - pig farmer	0.27	<0.001	0.239	<0.001
air - cow farmer	**0.964**	<0.001	**0.96**	<0.001
air - non-exposed	**1**	<0.001	**0.991**	<0.001
pig farmer - cow farmer	0.704	<0.001	**0.875**	<0.001
pig farmer - non-exposed	**0.756**	<0.001	**0.968**	<0.001
cow farmer - non-exposed	0.314	<0.001	**0.814**	<0.001

[a] Values printed in bold represent highly different groups (0.75<R)

Supplementary table S2: Significant SVs according to the ‘abundance based’ approach, presence/absence analysis and the ANOVA-Like Differential Expression (ALDEx) Analysis

SVs significantly associated in abundance approach but neither in presence/absence nor ALDEx approach (n=1)	SV125
SVs significantly associated in ALDEx approach but neither in presence/absence nor abundance approach (n=9)	SV133, SV195, SV227, SV431, SV450, SV473, SV567, SV596, SV668
SVs significantly associated in presence/absence approach but neither in ALDEx nor abundance approach (n=5)	SV216, SV317, SV334, SV372, SV400
SVs significantly associated in abundance and presence/absence approach but not in ALDEx approach (n=40)	SV13, SV39, SV53, SV70, SV90, SV94, SV111, SV141, SV143, SV149, SV159, SV162, SV183, SV184, SV190, SV193, SV202, SV222, SV228, SV233, SV236, SV238, SV254, SV260, SV265, SV279, SV285, SV297, SV302, SV303, SV325, SV327, SV350, SV358, SV368, SV376, SV424, SV476, SV533, SV547
SVs significantly associated in all three approaches (abundance, presence/absence and ALDEx) (n=41)	SV3, SV5, SV7, SV14, SV15, SV17, SV19, SV20, SV21, SV23, SV35, SV36, SV38, SV43, SV48, SV56, SV57, SV59, SV63, SV69, SV78, SV81, SV83, SV84, SV91, SV107, SV109, SV119, SV122, SV130, SV135, SV153, SV163, SV170, SV198, SV209, SV213, SV223, SV251, SV284, SV298

Figure legends of supplementary figures

Figure S1. Rarefaction curves of all the samples included in this study (n=255). **A** pig (n=56), **B** air (n=27), **C** pig farmer anterior and posterior (n=86), **D** cow farmer anterior and posterior (n=34), **E** non-exposed anterior and posterior (n=52)

Figure S2. Effect plots summarizing the ALDEx2 output. Illustrated are the comparisons of **A)** pigs versus cow farmers, **B)** air versus cow farmers and **C)** pig farmers versus cow farmers. In these plots, each point represents an individual SV from the data set with the expected value of the log2 difference between groups on the y-axis and the expected value of the maximum within-group dispersion on the x-axis. Thus, the location each point in the plot provides a graphic summary of the standardized difference-dispersion relationship for each SV. SVs with BH-corrected p values less than or equal to 0.05 are shown in red and SVs with BH-corrected p values more than 0.05 are shown in grey. The 82 SVs that were identified as significant in the presence/absence and abundance approach are green-rimmed. Diagonal lines are shown for zero-intercept lines with slopes of ±1 and ±2, and these lines correspond to the expected location of points with the corresponding effect sizes.

Figure S3. Venn diagram of the three different anaylses. Significant SVs according to the 'abundance based' approach, presence/absence analysis and the ANOVA-Like Differential Expression (ALDEx) Analysis

Figure S4. Sequence variants (SVs) associated with pig farming and differential SVs between anterior and posterior nasal samples. Illustrated are the 10 most abundant SVs (ordered from most abundant to least abundant). Shown are **A)** the heatmaps depicting relative abundances and frequencies for pig (n=56), air (n=27), pig farmer (n=56), cow farmer (n=17) and non-exposed (n=26). Assigned taxonomy (bacterial genus, order or family) for each SV is shown, too. The **B)** Forest plot displays the coefficients of pairwise differences between anterior and posterior nasal samples from pig farmers derived by wilcoxon singed rank tests followed by Benjamini-Hochberg correction. Significant differences after multiple testing are illustrated (*)

Figure S5. Taxonomic profile comparison with taxa assignment based on DADA2 and mothur pipelines for all sample types. Shown are **A)** the mean relative abundance of phyla based on for DADA2, **B)** the mean relative abundance of families based on DADA2, **C)** the mean relative abundance of phyla based on mother and **D)** the mean relative abundance of families based on mothur

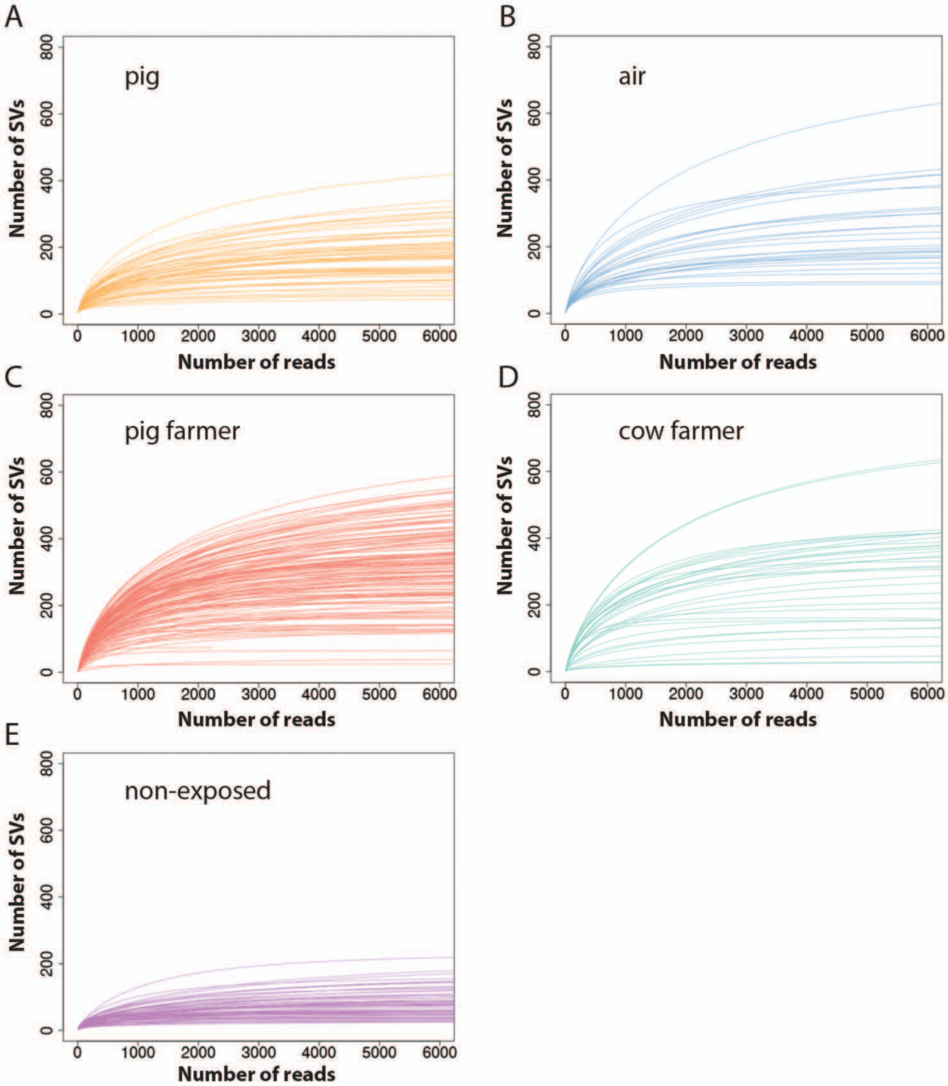

A
pig
B
air
C
pig farmer
D
cow farmer
E
non-exposed
Number of SVs
Number of reads
0
200
400
600
800
0
1000
2000
3000
4000
5000
6000

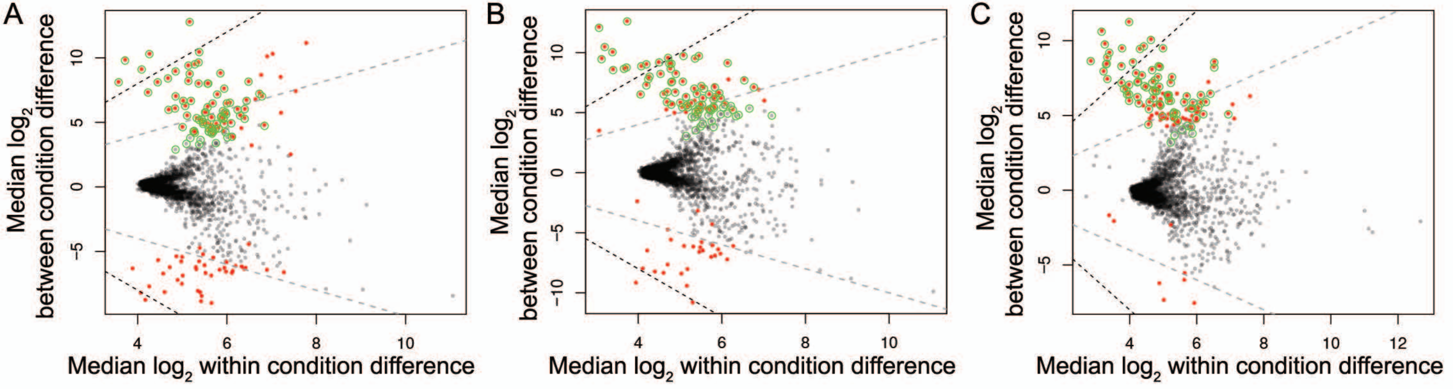

A
B
C
Median $\log_2$ between condition difference
Median $\log_2$ within condition difference

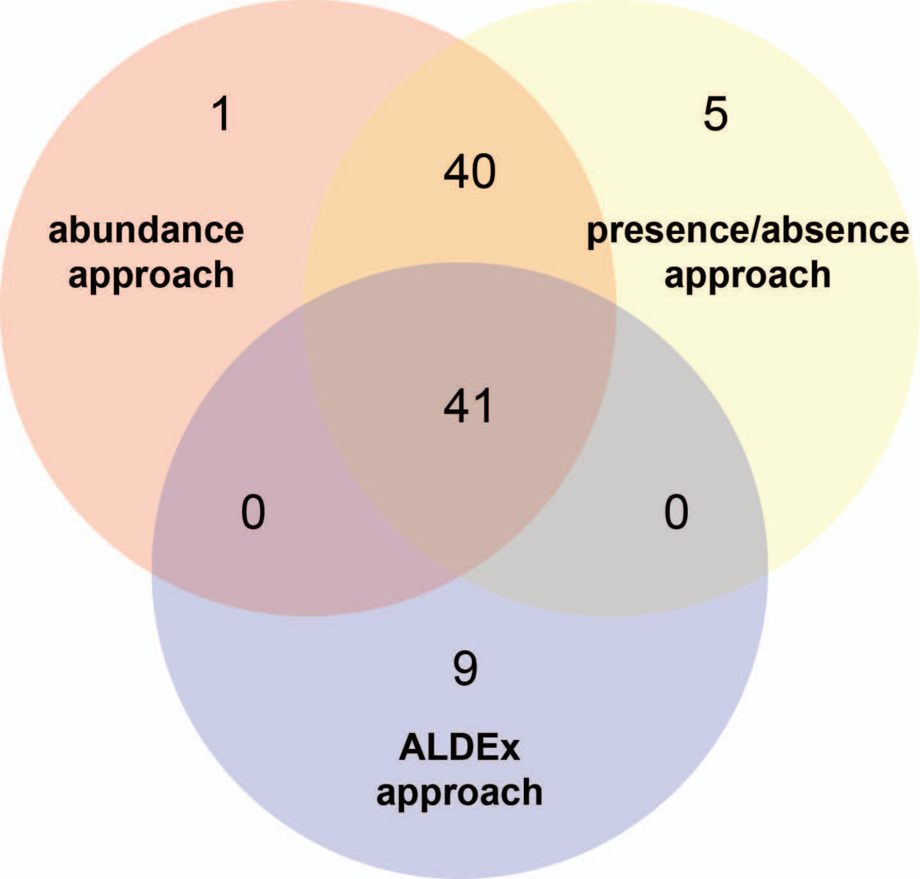
1
40
5
abundance
approach
presence/absence
approach
41
0
0
9
ALDEx
approach

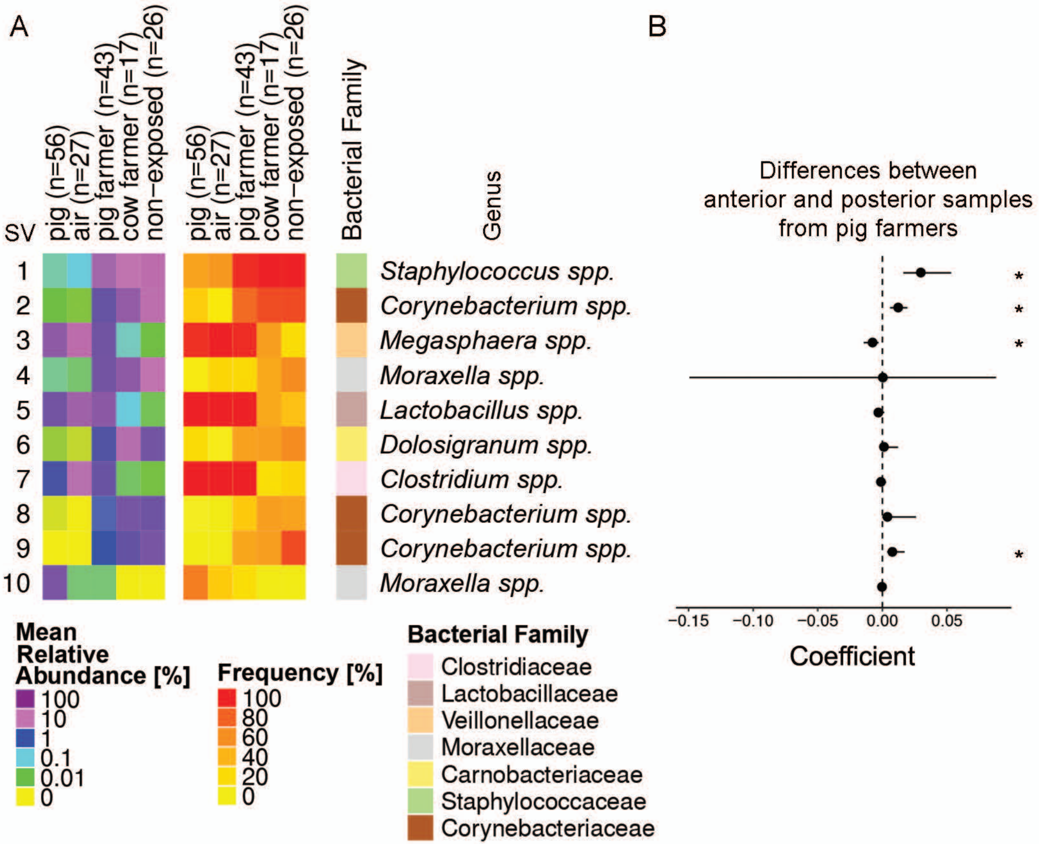
A
SV
pig (n=56)
air (n=27)
pig farmer (n=43)
cow farmer (n=17)
non−exposed (n=26)
pig (n=56)
air (n=27)
pig farmer (n=43)
cow farmer (n=17)
non−exposed (n=26)
Bacterial Family
Genus
1
2
3
4
5
6
7
8
9
10
Staphylococcus spp.
Corynebacterium spp.
Megasphaera spp.
Moraxella spp.
Lactobacillus spp.
Dolosigranum spp.
Clostridium spp.
Corynebacterium spp.
Corynebacterium spp.
Moraxella spp.
Mean Relative Abundance [%]
100
10
1
0.1
0.01
0
Frequency [%]
100
80
60
40
20
0
Bacterial Family
Clostridiaceae
Lactobacillaceae
Veillonellaceae
Moraxellaceae
Carnobacteriaceae
Staphylococcaceae
Corynebacteriaceae
B
Differences between anterior and posterior samples from pig farmers
*
*
*
*
−0.15
−0.10
−0.05
0.00
0.05
Coefficient

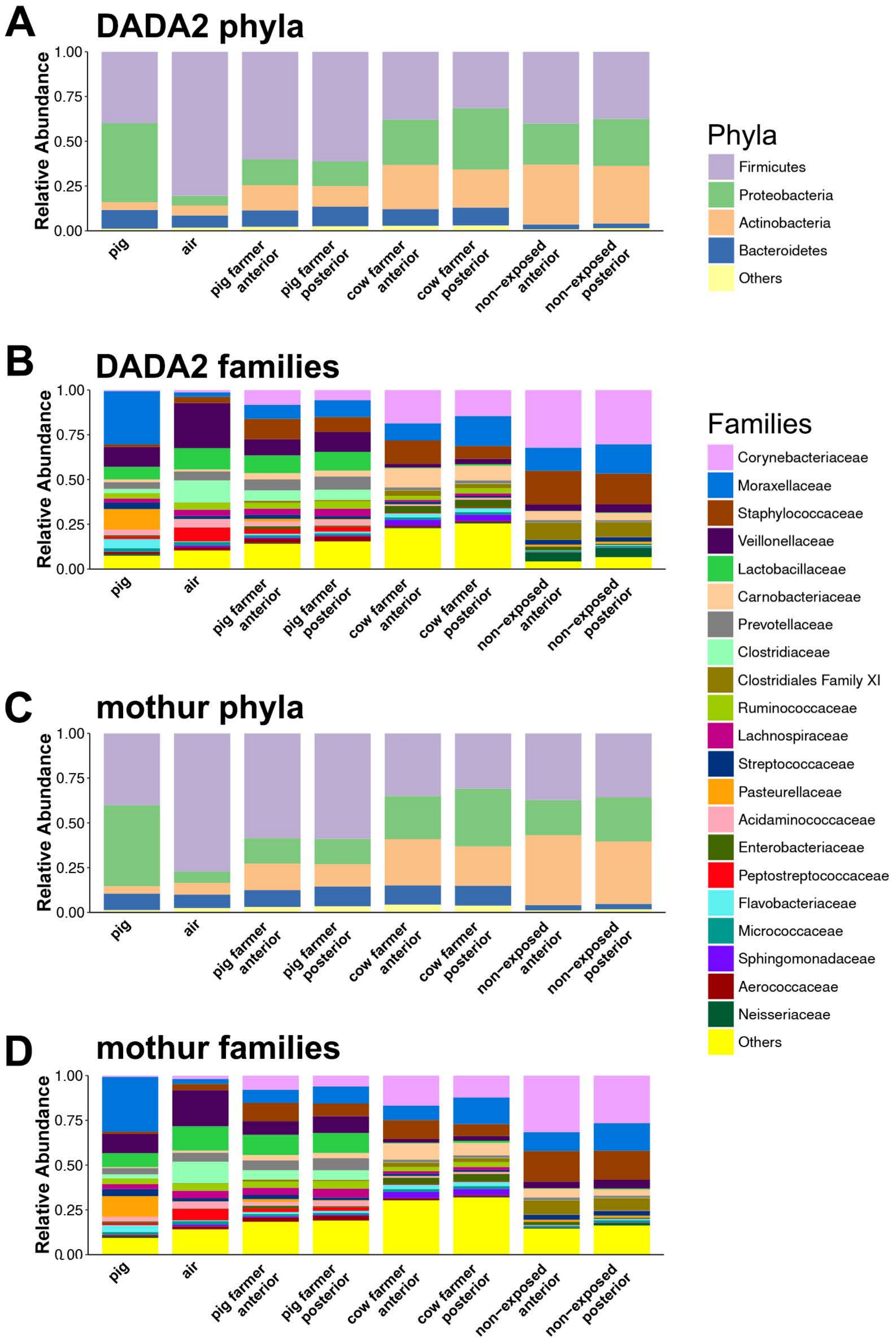
A
DADA2 phyla
B
DADA2 families
C
mothur phyla
D
mothur families
Relative Abundance
0.00
0.25
0.50
0.75
1.00
pig
air
pig farmer anterior
pig farmer posterior
cow farmer anterior
cow farmer posterior
non-exposed anterior
non-exposed posterior
Phyla
Firmicutes
Proteobacteria
Actinobacteria
Bacteroidetes
Others
Families
Corynebacteriaceae
Moraxellaceae
Staphylococcaceae
Veillonellaceae
Lactobacillaceae
Carnobacteriaceae
Prevotellaceae
Clostridiaceae
Clostridiales Family XI
Ruminococcaceae
Lachnospiraceae
Streptococcaceae
Pasteurellaceae
Acidaminococcaceae
Enterobacteriaceae
Peptostreptococcaceae
Flavobacteriaceae
Micrococcaceae
Sphingomonadaceae
Aerococcaceae
Neisseriaceae
Others
